# Motor and cognitive determinants of L2 handwriting fluency and legibility in 10–12-year-old girls: a cross-sectional study

**DOI:** 10.3389/fpsyg.2026.1757652

**Published:** 2026-04-23

**Authors:** Mohamed Frikha, Zainab Ali Alsalman, Mehdi Chlif

**Affiliations:** 1 Department of Physical Education, College of Education, King Faisal University, Al-Ahsa, Saudi Arabia; 2 Research Laboratory: Sports Performance Optimisation, National Censter of Medicine and Science in Sports (NCMSS), Tunis, Tunisia; 3 EA 3300 “APS and Motor Patterns: Adaptations-Rehabilitation”, Picardie Jules Verne University, Amiens, France

**Keywords:** cross-sectional study, executive functions, fluency, handwriting, kinesthetic differentiation, legibility, manual dexterity, motor skills

## Abstract

**Background:**

Handwriting remains an essential academic skill even with the rising use of digital tools in classrooms settings. This cross-sectional observational study investigated the interplay between motor and cognitive determinants in the second-language (L2) handwriting fluency and legibility among typically developing school-aged girls.

**Methods:**

L2 is operationalised via English-medium schooling exposure among Arabic L1 speakers, and English proficiency was not directly assessed. A total of 129 girls, aged 10–12 years, were asked to complete the Handwriting Speed Test (HST; letters per minute), and three experienced English teachers were invited to assess the Legibility using the Handwriting Legibility Scale (HLS; the higher the score, poorer the legibility). Measures of predictor include; manual dexterity (Grooved Pegboard Test), motor speed (Finger-tapping test over 10 and 30s), kinesthetic differentiation (force-production error in a grip-matching task), executive flexibility (Trail-making Test Part B), and visuospatial working memory (Corsi block-tapping span). Multiple linear regression models were estimated using simultaneous entries.

**Results:**

Handwriting fluency was independently predicted by better manual dexterity, higher sustained motor speed, better executive flexibility, and greater visuospatial working memory, together explaining 52% of the variance in HST scores (*R*^2^ = 0.520, *p* < 0.001). Reduced legibility was also independently correlated with lower manual dexterity, higher reliability in the kinesthetic error and lower executive flexibility that explained 47% of the variance in the HLS scores (*R*^2^ = 0.468, *p* < 0.001). A modest inverse correlation between fluency and legibility (*r* = −0.27, *p* < 0.01) indicated a speed–legibility trade-off.

**Discussion:**

These findings suggest that L2 handwriting fluency depends on efficient and sustained motor output supported by executive control and working memory, whereas legibility relies more on fine motor precision and proprioceptive accuracy, with executive flexibility emerging as a shared correlate across both handwriting dimensions. Targeted interventions that combine fine-motor and kinesthetic training with executive-function support may help optimise both speed and legibility in classroom settings.

## 1 Introduction

Despite the growing incorporation of digital devices in educational settings, handwriting remains a fundamental skill in classrooms, essential for note-taking, timed assessments, and written composition throughout primary and secondary education (Araújo et al., 2022; Bonafede and van der Merwe, 2023; Bonneton-Botté et al., 2023; Haleem et al., 2022). Compared to typing, handwriting offers better cognitive and memory advantages, especially in the academic and learning contexts (Marano et al., 2025). Handwriting is generally understood as an integrated cognitive-motor skill, wherein lower-level perceptual-motor processes interact with higher-level executive and mnemonic controls (Graham and Weintraub, 1996; Rosenblum et al., 2019). The skills in handwriting are usually distinguished into fluency, and legibility (Frikha et al., 2025; Kent et al., 2014). Legibility involves, among other, the formation of letters, letter size, letter spacing, and letter alignment, and fluency concerns the speed and fluency of writing (Vico et al., 2023; Rosenblum et al., 2003). Non-parallel developmental patterns have been shown in research, and legibility has an acceptable level at earlier ages than speed, especially in later years of primary education (Skar et al., 2023; Gosse et al., 2021; Wallen et al., 2006).

Previous studies (Potgieser et al., 2015; Semeraro et al., 2019), highlighted that the cognitive demands linked to language familiarity may modulate the patterns of brain activation that occur during the act of handwriting, thereby providing valuable insights into the relationship between language, cognition, and motor skills. Nonetheless, little is known about most of the co-occurring processes (i.e., concurrent cognitive and motor processes required during handwriting and transcription) that a child must engage in when handwriting in a second language (L2). Moreover, uncertainty remains regarding which factors contribute to individual differences in L2 handwriting fluency and legibility under classroom-like time constraints (Kormos, 2023). In this context, L2 is operationalised via English-medium schooling exposure rather than direct proficiency testing; therefore, our claims are limited to handwriting performance observed during English-language classroom demands.

### 1.1 Cognition, and motor skills in L1 versus L2 handwriting

According to the hierarchical model of van Galen (1991), Afonso et al. (2018) shifted the emphasis on the interaction between the higher-level linguistic-based processes and the lower-level motor execution when it comes to the L1 handwriting. The authors further posit that the orthographic retrieval comes before the motor actions take place, and that the central effects are often produced before the handwriting is activated. This view offers a model that can be used to explain the difference between the native and second language handwriting, in which the requirements of lexical access and orthographic retrieval can trigger different cognitive loads and hence affect motor performance. From a developmental perspective, it would mean that handwriting becomes a skill that does not rely on the lexical information to a great extent, around the age of eleven years (Afonso et al., 2018).

Recently, Maurer et al. (2023) emphasize the importance of handwriting for developing literacy skills. The authors suggested that the graphomotor skills interplay with orthographic knowledge, working memory, visuospatial skills and executive functioning, thus determining the overall native versus L2 handwriting in the early years of schooling.

Besides, findings suggest that handwriting development differs between girls and boys, not only in the final product but also in the underlying skills that lead to legible, fluent, and efficient writing. Significant gender differences were observed, with girls outperforming boys in fine motor skills, visuomotor integration, and the legibility of their handwriting (Maurer, 2024). Similar findings were observed by, Yang et al. (2020), investigating the neural basis of sex differences in handwriting using fMRI, and suggesting that men and women utilize partially distinct neural pathways to achieve handwriting, and that women producing higher-quality of handwriting than men.

Despite the lack of empirical studies of the comparison between L1 and L2 handwriting, the available results support the assumption that handwriting activates specific cognitive-motor networks that are not familiar with writing in L2. Such discrepancy shows the possible age- and modality-related differences in cognitive load, affecting the results of learning (Ose Askvik et al., 2020). Based on the fact that L2 handwriting refers to a new writing system for learners (new symbols, rules, punctuation, script, orthographic convention), it appears necessary to develop new literacy skills or adapting the pre-existing ones (Cook and Bassetti, 2005). In a review, (Altheneyan and Boayrid, 2019), it was highlighted the transfer of native Arabic (L1) in English (L2) writing, by the well-known process of language interference (Odlin, 2003). This transfer perspective also suggests that part of the variability observed in English handwriting may originate from previously established Arabic L1 graphomotor routines. Cross-script evidence indicates that some handwriting skills can transfer across alphabets, while biscriptual experience can also reshape graphomotor coordination and fine-motor control in script-specific ways (Asselborn et al., 2021; Alhaddad et al., 2023; Alhaddad et al., 2024).

Research indicates that children are different from adults as they acquire L2 (Lopez and Vaivre-Douret, 2021), and that L2 handwriting performance is highly related to executive function that depends on language proficiency and task demands (Haake, 2025). Thus, understanding how distinct motor and cognitive systems support L2 handwriting fluency and legibility is crucial for theoretical understanding and classroom practice.

In the motor domain, three constructs are commonly related to handwriting; these are manual dexterity, motor speed and kinesthetic differentiation. Assessed with the help of tests (including the Grooved Pegboard Test) and used to measure the dexterity of the hands, it enables the efficient in-hand control and quick changes of strokes (Giustino et al., 2023). Motor speed, typically assessed via finger tapping tasks, provides an index of maximal repetitive motor tempo that may constrain—but does not fully represent—the continuous, feedback-driven motor control required in handwriting (Schatz, 2011; Heimhofer et al., 2024). Kinesthetic differentiation, measured by force production error in matching a target grip force, reflects proprioceptive–motor calibration; because grip-force matching requires both proprioceptive input and motor command generation, we interpret force production error as relevant to pen pressure regulation and fine-motor precision in handwriting (Prunty et al., 2020; Raczek et al., 2003). Although each of these elements has been associated with written performance, other studies have not examined them concurrently in the same model to determine their individual and combined role in determining handwriting speed and legibility.

In cognitive science, the executive functions, specifically set shifting/flexibility and the visuospatial working memory are commonly perceived in the context of handwriting proficiency and literacy outcomes. Our selection of executive constructs was guided by Diamond’s (2013) framework, which identifies working memory, inhibition, and cognitive flexibility as core executive functions; in the present study, we focused on working memory and cognitive flexibility because they are especially proximal to transcriptional demands such as temporary information maintenance, set shifting, monitoring, and online correction during handwriting, whereas inhibition was not directly assessed. The executive flexibility that is usually measured through the Trail-Making Test Part A and B improves quick shifting between cognitive tasks, monitoring of current tasks, as well as corrective actions while acting within time limitation (Diamond, 2013; Reitan and Wolfson, 2001). Visuospatial working memory, typically assessed through tasks such as the Corsi Block-Tapping span, is posited to support spatial layout demands (e.g., spacing, alignment) and the temporary maintenance of visuospatial sequences during handwriting and transcription (Corsi, 1973; McCutchen, 1996; Vasylets and Marín, 2021). McCutchen (1996) is cited here as support for the broader claim that writing draws on limited working-memory resources, rather than as evidence for a specifically visuospatial subsystem. Recent studies suggest that limitations in executive and working memory may constrain transcription skills and that automatising lower-level processes could liberate resources for higher-level planning and composition (Medwell and Wray, 2014; Downing and Caravolas, 2023; McCutchen, 1996; Salas and Silvente, 2020). Nevertheless, the relative and combined contributions of fine motor abilities, proprioception, executive flexibility, and visuospatial working memory to handwriting speed and legibility remain insufficiently characterised in typically developing girls.

### 1.2 The trade-off between speed and legibility

A modest trade-off between speed and legibility is frequently discussed in the handwriting literature. Gosse et al. (2021) is used more specifically here to support the non-parallel developmental trajectories of handwriting speed and handwriting quality during primary school, rather than as direct evidence of a behavioral trade-off. Likewise, Planton et al. (2013) does not directly test a speed–quality trade-off; instead, it supports the idea that motor execution and orthographic/spatial processes in handwriting are partially dissociable. Theories of motor control in handwriting (van Galen, 1991) and neuroimaging evidence (Planton et al., 2013) converge on the notion that stroke timing (temporal control) and stroke shape (spatial control) are partially dissociable processes coordinated by executive systems under temporal constraints. From an applied perspective, this suggests that augmenting speed without adequate automatisation may overburden monitoring resources and impair legibility (Feder and Majnemer, 2007; Santangelo and Graham, 2016). However, much of the extant empirical research either concentrates on global handwriting proficiency scores or examines speed and legibility separately, often employing limited predictor sets (e.g., fine motor skills alone) or broad age ranges that obscure developmental patterns. Furthermore, kinesthetic differentiation is seldom included along with dexterity and motor speed, and relatively few studies have employed multivariable models to distinguish between unique and shared variances in late primary school samples.

### 1.3 The present study

To minimise developmental confounds and enhance interpretability for educational practice, we focused on a specific age range of 10–12 years and concentrated on female participants who typically exhibit slightly earlier maturation in fine motor and literacy skills (Feder and Majnemer, 2007; Vaivre-Douret et al., 2021). This late primary window was selected because legibility is typically established whereas speed continues to develop, allowing meaningful modelling of individual differences in fluency. Employing validated measures—the Handwriting Speed Test (HST; Wallen et al., 2006) and the Handwriting Legibility Scale (HLS; Barnett et al., 2018; higher HLS scores denote poorer legibility)—we implemented a multidomain predictor battery. This battery included the Grooved Pegboard Test (manual dexterity), Finger Tapping Test at 10 s and 30 s (motor speed), force production error in a grip-matching task (kinesthetic differentiation), Trail Making Test Part B (executive flexibility), and Corsi Block-Tapping span (visuospatial working memory) (Wilcox and Nordstokke, 2022). We nevertheless acknowledge that meaningful developmental differences in transcription may still exist within the 10–12-year band.

In view to the above considerations, we posited that L2 handwriting fluency (HST) in this English-medium schooling context is independently correlated with superior manual dexterity, enhanced sustained motor speed, increased executive flexibility, and improved visuospatial working memory. Conversely, we anticipated that poorer legibility (higher HLS) would be independently associated with diminished dexterity, increased kinesthetic errors, and reduced executive flexibility. Additionally, we expected a modest inverse correlation between speed and legibility, aligning with a partial trade-off rather than strict opposition (Connelly et al., 2005; Sumner et al., 2012; Gosse et al., 2021).

## 2 Materials and methods

### 2.1 Research design and sample size calculation

Using a mixed methods involving students’ performances and experts’ assessments, this cross-sectional observational study examined the contribution of specific motor and cognitive factors to L2 handwriting speed and legibility in typically developing school-girls. Handwriting performances were operationalised through two complementary dimensions: fluency, which refers to speed of production and legibility which refers to the clarity and accuracy of the written output. The predictor variables were selected to represent well-elaborated areas of interest to handwriting, which are namely manual dexterity, motor speed, kinesthetic differentiation, executive flexibility and visuospatial working memory. All tests were conducted separately, following the regular procedures, in a quite school setting, in accordance with the recommendations for observational behavioral research in educational contexts. A preliminary estimate of the sample size was calculated through G*Power (version 3.1.9.2). Using a medium effect size of 0.15, an alpha level of 0.05 and a required statistical power of 0.95, a recommended sample size of 89 participants was determined. Considering a possible dropout rate, 129 participants were temporarily selected and invited to the familiarisation sessions.

### 2.2 Participants

#### 2.2.1 Inclusion- exclusion criteria

The sampled group (129 girls aged 10–12 years old) was recruited by a convenience sampling method from Al-Reeyada International School (ARIS). Restricting the age range to late childhood aimed to reduce the heterogeneity in developmental stages of handwriting maturation and neurocognitive performance. Also, the restriction of participation to girls only is related to the previously gender differences that were observed, with girls outperforming boys in fine motor skills, visuomotor integration, and the legibility of their handwriting (Maurer, 2024), and to gender distinct neural pathways that were highlighted, suggesting that men and women utilize partially distinct neural pathways to achieve handwriting (Yang et al., 2020). The potential participant had to be typically developing and be attending mainstream school, and have no neurological, orthopaedic, psychiatric or uncorrected sensory problems that would interfere with their performance. Children who had a history of learning problems, previously that were specific to writing or handwriting were excluded. Brief laterality screening was used in recording hand preference. Written parental consent and child assent were obtained prior to testing. All procedures adhered to the Declaration of Helsinki and were approved by the Research Ethics Committee of King Faisal University (KFU-REC-2023-JUN-ETHICS988). Thus, age-range restriction was used as a design-based control to reduce heterogeneity, but it was not equivalent to statistically modelling age-related variance.

#### 2.2.2 Participants cultural background

All participants shared a homogeneous social background, and were native Arabic speakers. They had been attending ARIS for a minimum of five to six years. This institution delivers its core curriculum exclusively in English following the British system. The English-medium instruction encompasses daily classes in English Language, Mathematics, Social Studies, ICT, and Science. Additional subjects are taught with varying frequency: Islamic Culture and Physical Education are offered three times and once per week, respectively, while only Arabic Language and Deen (in Arabic too) are taught five times and twice per week, respectively. ARIS holds accreditation as an international examination center from Cambridge University and is attached to the British Council. Furthermore, the school is accredited by AdvancED, a global American accreditation agency. Accordingly, the present sample reflects an English-dominant Arabic-English biscriptual educational context rather than fully balanced exposure to the two languages/scripts.

### 2.3 Outcome measures

*Handwriting Fluency* was evaluated using the Handwriting Speed Test (HST), which required participants to continuously copy a standardised pangram for 1 min, in accordance with the original protocol established by Wallen and Mackay (1999). The primary outcome measure, letters per minute, represents the number of legible letters produced, with higher values indicating greater fluency. Administration instructions and scoring were strictly adhered to as per the published manual to ensure comparability with previous studies.

*Handwriting Legibility* was evaluated using the Handwriting Legibility Scale (HLS), which assesses letter formation, spacing, size consistency, baseline alignment, and overall readability (Barnett et al., 2018). Three trained examiners independently scored the anonymised handwriting samples. Higher scores indicated poorer legibility. Inter-rater reliability was determined using a two-way random-effects intraclass correlation coefficient (ICC) based on absolute agreement, with 95% confidence intervals calculated for the ICC values. Scoring discrepancies exceeding one point were resolved through a consensus review following a joint examination of the samples, with raters remaining blinded to all motor and cognitive data.

### 2.4 Predictor measures

*The manual dexterity* was evaluated using the *Grooved Pegboard Test* (GPT; Lafayette Instrument Company), which required participants to swiftly insert keyed pegs into slotted holes using their dominant hands. The primary metric was the time taken to complete the task, recorded in seconds, with shorter times indicating enhanced dexterity (Merker and Podell, 2011). The psychometric properties of the GPT have been comprehensively documented in recent literature (Tolle et al., 2020; Giustino et al., 2023), and the standard procedures for administration and scoring, as detailed in these sources, were meticulously followed.

*The motor speed* was evaluated utilising the Finger Tapping-Test (FTT) in both 10-s (burst) and 30-s (sustained) intervals to capture short-burst and continuous motor output pertinent to classroom handwriting requirements. The FTT was executed using the Speedy Tapper application (Version 2.3.2), which was installed on a digital touchscreen device (Apple iPadOS 17.6.1, Retina, 10.2-inch screen).^1^ The results were quantified as the number of taps per interval, with higher scores denoting increased motor speed, in accordance with the administration protocols described by Schatz (2011) and Heimhofer et al. (2024). Participants were instructed to tap “as fast as possible without stopping” using the index finger of the dominant hand, and practice trials were provided as necessary to ensure comprehension of the task. Because the Speedy Tapper application returned tap counts only, no spatial accuracy index (e.g., tap dispersion or touch-location variability) could be extracted from this task. The FTT should therefore be interpreted here as an index of motor tempo rather than graphomotor spatial precision.

*The kinesthetic differentiation* was assessed through a blindfolded grip-force matching task, wherein children endeavoured to replicate a target grip force (50% of the maximal force). The participants were directed to sit on a chair throughout the testing process, with the forearm of the right arm maintained in a neutral position and the elbow joint flexed at approximately 90° (Mathiowetz et al., 1986). A digital hydraulic dynamometer (Baseline BIMS, with an accuracy of 1%) was used to measure grip strength. Performance was quantified as the percentage deviation between the exerted force and target value, utilising the formula proposed by Raczek et al. (2003):


FPE=∣target−produced∣/target×100.

Lower percentage deviation values signified more precise proprioceptive control. The target force level was standardised among the participants, and practice attempts were made to ensure understanding of the procedure.

*The executive flexibility* was evaluated using the Trail-Making Test (TMT). The test is composed of two parts A and B. In the first part A, the participant is asked to follow an ascending order (1-2-3-4…) of 25 circles randomly distributed on the screen, according to the numbers written inside of each. It starts with the number 1 and ends with 25. In the second part B of the task, the participant has to do the same thing, but this time they have to replace the numbers in ascending order and the letters in alphabetical order (1-A, 2-B…), starting with 1 and ending with 13. For both parts, we looked at how quickly participants could complete the tasks, which measured their visual scanning and psychomotor speed. Generally, quicker completion times suggest better performance (Reitan and Wolfson, 2001). The test was carried out using a digital touchscreen device (an Apple iPad running iPadOS 17.6.1, with a Retina display and a screen size of 10.2 inches), through the “Trail Making Exam J” app (Version 1.2). This digital approach offers several advantages over traditional paper-based methods, including standardised administration, precise timing evaluations, and reduced examiner bias (Simfukwe et al., 2020).

*The visuospatial working memory* was evaluated using the forward span of the Corsi Block Tapping Test (CBT). The primary measure was the longest sequence accurately reproduced, following the original methodology outlined by Corsi (1973). The sequences gradually extend in length, mirroring the conventional approach, beginning with a sequence of two squares and subsequently advancing to nine squares (Richardson, 2007). In the present study, only five sequences were given to participants (from three to seven), which were repeated two times. In sum, 10 attempts were made, and the number of correct attempts was retained. The test was performed online^2^.

### 2.5 Procedures

Testing was conducted in quiet indoor classrooms during standardised morning sessions to minimise the impact of fatigue or diurnal variations. After obtaining consent, assent, and completion of eligibility screening, handwriting tasks (HST and HLS sample collection) were administered to prevent potential motor fatigue effects. Subsequently, the participants undertook motor and cognitive assessments. The sequence of predictor measures was systematically counterbalanced among participants to mitigate order and fatigue effects. Short rest periods were provided between tasks as needed. All assessments were administered by the same trained examiner using standardised written scripts and demonstration trials to ensure procedural consistency. Handwritten samples were anonymised prior to scoring, and raters were blinded to the participants’ motor and cognitive results to prevent expectancy bias. Data collection was completed within a single session per participant, with an approximate duration of 45–60 min. A fully randomized task order was not used; we chose counterbalancing to preserve a consistent testing flow while still reducing order and fatigue effects, although future studies may compare counterbalanced, fully randomized, and more ecologically sequenced task orders.

### 2.6 Data handling

Data management adhered to the established scoring protocols specific to each instrument. For the Grooved Pegboard Test, the fastest completion time using the dominant hand was selected when two trials were conducted. In the case of the Finger Tapping Test, if multiple runs were performed, the average number of taps across valid trials was recorded. Scores for the Handwriting Speed Test were determined by the number of legible letters written per minute, whereas Handwriting Legibility Scale scores were calculated by summing ratings across various domains, with higher total scores indicating reduced legibility. The inter-rater reliability of the Handwriting Legibility Scale was assessed using ICC (2, 1) with 95% confidence intervals, as previously mentioned. All scoring procedures were consistent with published manuals and methodological literature (Barnett et al., 2018; Corsi, 1973; Giustino et al., 2023; Heimhofer et al., 2024; Raczek et al., 2003; Reitan and Wolfson, 2001; Schatz, 2011; Tolle et al., 2020; Wallen et al., 2006). Data were anonymised prior to analysis, and double-entry checks were implemented to minimise transcription errors.

### 2.7 Data analysis

Statistical analyses were conducted using IBM SPSS Statistics, version 26. Descriptive statistics are presented as means and standard deviations. The normality of residuals for the regression models was assessed using the Shapiro–Wilk test in conjunction with an examination of quantile–quantile plots, while homoscedasticity and linearity were evaluated through residual diagnostics. Pearson’s correlation coefficients were used to describe bivariate associations between outcomes and predictors. Two multiple regression models were computed using simultaneous entries. In the first model, handwriting fluency (HST) was designated as the dependent variable; in the second model, handwriting legibility (HLS) served as the dependent variable. Predictor variables for both models included the Grooved Pegboard Test completion time, 10-s and 30-s Finger Tapping Test performance, force production error, Trail Making Test Part B completion time, and Corsi span. Although the Trail Making Test Part A was initially considered a covariate, it was retained in the models for comprehensive reporting, despite not emerging as an independent predictor. Multicollinearity was assessed using tolerance and variance inflation factor values, and influential observations were evaluated using Cook’s distance and studentized residuals. No cases exceeded the conventional influence thresholds. Significance was set at *p* < 0.05 (two-tailed). Reporting included unstandardised and standardised coefficients, 95% confidence intervals, partial correlations, and explained variance, in accordance with recommended practices for multivariable behavioral modelling in school-aged samples and APA 7th-edition statistical reporting guidelines. Age was not entered as a separate covariate because the sample was intentionally restricted to a narrow late-primary developmental band; however, this design choice does not eliminate residual within-band developmental variability.

## 3 Results

### 3.1 Descriptive statistics

All 129 participants completed the full assessment battery without missing data. Table 1 summarises the descriptive statistics for the handwriting outcomes and motor–cognitive predictors. Handwriting speed (HST) averaged 99.64 (SD = 20.98) letters·min^−1^, indicating substantial inter-individual variation in fluency. Handwriting legibility (HLS; higher scores = poorer legibility) averaged 13.82 (SD = 4.57). Executive measures showed mean completion times of 55.31 (SD = 17.37) s for TMT-A and 106.73 (SD = 28.12) s for TMT-B, with corresponding 95% confidence intervals of [52.28, 58.34] for TMT-A and [101.83, 111.63] for TMT-B, reflecting moderate dispersion. Visuospatial working memory (Corsi Block-Tapping span) was 5.64 (SD = 2.51) blocks. Manual dexterity (Grooved Pegboard Test) averaged 133.59 (SD = 23.47) s, with lower values indicating better performance. Kinesthetic differentiation, indexed by force production error (FPE), had a mean of 15.10% (SD = 8.46). Motor speed measures were 5.56 (SD = 1.22) taps·s^−1^ for the 30-s Finger Tapping Test and 6.11 (SD = 0.71) taps·s^−1^ for the 10-s condition. These descriptive data indicated adequate variability across all domains for subsequent correlational and regression analyses (Table 1).

**Table 1:** Descriptive statistics of the dependents and independents variables (*N* = 129).

DV/IV	Mean	SD	Median	95% CI
				L–U
HST	99.637	20.979	103	95.982–103.292
HLS	13.820	4.565	13	13.025–14.616
TMT-A	55.310	17.367	55.7	55.310–61.361
TMT-B	106.734	28.115	102	101.836–11.632
CBT	5.643	2.511	6	5.206–6.081
GPT	133.589	23.471	130	129.5–137.678
FPE	15.095	8.458	13	13.621–16.568
FTT-30	5.560	1.224	5.57	5.346–5.773
FTT-10	6.107	0.712	6.2	5.982–6.231

HST, Handwriting Speed Test; HLS, Handwriting Legibility Scale; GPT, Grooved Pegboard Test; FPE, force production error; FTT, finger tapping test; TMT-A, trail-making test (A); TMT-B, trail-making test (B); CBT, Corsi-block test.

### 3.2 Bivariate associations

Pearson correlations among handwriting outcomes and predictors are presented in Table 2. As expected, HST and HLS were modestly and inversely related (*r* = −0.27, *p* < 0.01), indicating a small-to-moderate speed–legibility trade-off, whereby higher fluency tended to be associated with better legibility (lower HLS scores). Faster handwriting speed correlated with better manual dexterity (Grooved Pegboard, *r* = −0.48, *p* < 0.01), better executive performance (TMT-A: *r* = −0.43, *p* < 0.01; TMT-B: *r* = −0.38, *p* < 0.01), higher visuospatial working memory (Corsi span, *r* = 0.52, *p* < 0.01), and greater sustained motor speed (FTT-30, *r* = 0.44, *p* < 0.01). The short burst motor speed measure (FTT-10) showed a small, non-significant correlation with the HST (*r* = 0.15, *p* ≈ 0.09).

**Table 2:** Correlations between the dependant variables (HST, HLS), and the independent variables (GPT, FPE, FTT10, FTT30, TMT-A, TMT-B, and CBT) (*N* = 129).

DV/IV		HST	HLS	TMT-A	TMT-B	CBT	GPT	FPE	FTT30	FTT10
HST	Pear. correl	1								
	CI [95%]									
HLS	Pear. correl	−0.271^**^	1							
	CI [95%]	−0.424; −0.103								
TMT-A	Pear. correl	−0.433^**^	0.347^**^	1						
	CI [95%]	−0.563; −0.281	0.186; 0.491							
TMT-B	Pear. correl	−0.461^**^	0.575^**^	0.407^**^	1					
	CI [95%]	−0.587; −0.313	0.447; 0.680	0.252; 0.542						
CBT	Pear. correl	0.524^**^	−0.233^**^	−0.384^**^	−0.365^**^	1				
	CI [95%]	0.386; 0.639	−0.390; −0.063	−0.522; −0.226	−0.506; −0.205					
GPT	Pear. correl	−0.478^**^	0.477^**^	0.445^**^	0.350^**^	−0.345^**^	1			
	CI [95%]	−0.601; −0.332	0.331; 0.600	0.295; 0.574	0.189; 0.493	−0.489; −0.183				
FPE	Pear. correl	−0.185^*^	0.439^**^	0.192^*^	0.431^**^	−0.086	0.228^**^	1		
	CI [95%]	−0.347; −0.013	0.288; 0.569	0.020; 0.353	0.279; 0.562	−0.255; 0.089	0.057; 0.386			
FTT30	Pear. correl	0.442^**^	−0.194^*^	−0.246^**^	−0.159	0.253^**^	−0.150	−0.151	1	
	CI [95%]	0.291; 0.571	−0.355; −0.022	−0.402; −0.076	−0.323; −0.014	0.084; 0.408	−0.315; 0.023	−0.316; 0.022		
FTT10	Pear. correl	0.145	−0.072	−0.014	0.049	0.086	0.040	0.020	0.112	1
	CI [95%]	−0.028; 0.310	−0.241; 0.103	−0.187; 0.159	−0.125; 0.220	−0.088; 0.255	−0.134; 0.211	−0.153; 0.192	−0.062; 0.279	

HST, Handwriting Speed Test; HLS, Handwriting Legibility Scale; GPT, Grooved Pegboard Test; FPE, force production error; FTT, finger tapping test; TMT-A, trail-making test (A); TMT-B, trail-making test (B); CBT, Corsi-block test; ^*^Correlation is significant at the level *p* < 0.05 (2-tailed); ^**^Correlation is significant at the level *p* < 0.01 (2-tailed).

For legibility, poorer HLS scores (higher values) were associated with poorer manual dexterity (Grooved Pegboard, *r* = 0.48, *p* < 0.01), greater kinesthetic error (FPE, *r* = 0.44, *p* < 0.01), and poorer executive flexibility (TMT-B, *r* = 0.43, *p* < 0.01). Smaller associations were observed with the TMT-A (*r* = 0.19, *p* < 0.05) and FTT-30 (*r* = −0.19, *p* < 0.05). Visuospatial working memory showed a weak, non-significant association with HLS. Overall, these patterns suggest that handwriting fluency is more strongly related to sustained motor speed and working memory, whereas legibility is more closely related to dexterity, kinesthetic differentiation, and executive flexibility (Table 2).

### 3.3 Multivariable models

#### 3.3.1 Model 1: predictors of handwriting fluency (HST)

A multiple linear regression model predicting handwriting speed (HST) from manual dexterity (Grooved Pegboard), motor speed (FTT-10, FTT-30), kinesthetic differentiation (FPE), executive flexibility (TMT-B), visuospatial working memory (Corsi span), and TMT-A completion time accounted for a substantial proportion of variance, *R*^2^ = 0.520, adjusted *R*^2^ = 0.492, *F* (7, 121) = 18.74, *p* < 0.001 (Table 3, Panel A). In this multivariate context, four predictors emerged as independent correlates of fluency. Better manual dexterity (shorter Grooved Pegboard times) was associated with faster handwriting [*B* = − 0.226, 95% CI (−0.356, −0.096), *t* = −3.44, *p* < 0.001]. Higher sustained motor speed (FTT–30) predicted higher HST scores [*B* = 4.836, 95% CI (2.571, 7.101), *t* = 4.23, *p* < 0.001]. Better executive flexibility (shorter TMT-B completion times) was independently related to faster handwriting [*B* = −0.176, 95% CI (−0.292, −0.060), *t* = −3.01, *p* = 0.003]. Finally, higher visuospatial working memory (Corsi span) was associated with greater fluency [*B* = 2.068, 95% CI (0.856, 3.280), *t* = 3.38, *p* < 0.001].

**Table 3:** Multiple linear regression between the outcome variables (HST, HLS), and the predictors variables (GPT, FPE, FTT10, FTT30, TMT-A, TMT-B, and CBT) (*N* = 129).

DV			*β* (SE)	*R* ^2^	*t*	Sig.	95% CI for *β*
							L–U
Panel A	HST	Constant (*N* = 129)	92.813 (15.918)	0.520	5.831	<0.001***	61.299–124.327
		GPT	−0.226 (0.066)		−3.444	<0.001***	−0.356–−0.096
		FPE	0.121 (0.176)		0.687	0.494	−0.227–0.469
		FTT10	3.304 (1.882)		1.756	0.082	−0.422–7.030
		FTT30	4.836 (1.144)		4.227	<0.001***	2.571–7.101
		TMTa	−0.081 (0.092)		−0.881	0.380	−0.263–0.101
		TMTb	−0.176 (0.059)		−3.009	0.003**	−0.292–−0.060
		CBT	2.068 (0.612)		3.378	<0.001***	0.856–3.280
Panel B	HLS	Constant (*N* = 129)	1.811 (3.648)	0.468	0.496	0.620	−5.410–9.032
		GPT	0.059 (0.015)		3.888	<0.001***	0.029–0.088
		FPE	0.105 (0.040)		2.607	0.010**	0.025–0.185
		FTT10	−0.678 (0.431)		−1.573	0.118	−1.532–0.176
		FTT30	−0.205 (0.262)		−0.782	0.436	−0.724–0.314
		TMTa	0.006 (0.021)		0.301	0.764	−0.035–0.048
		TMTb	0.064 (0.013)		4.795	<0.001***	0.038–0.091
		CBT	0.116 (0.140)		0.828	0.410	−0.162–0.394

HST, Handwriting Speed Test; HLS, Handwriting Legibility Scale; GPT, Grooved Pegboard Test; FPE, force production error; FTT, finger tapping test; TMT-A, trail making test (A); TMT-B, trail making test (B); CBT, Corsi-block test; *β*, unstandardized coefficient; SE, standard errors; DV, dependent variable; *Significantly different at: **p < 0.01; ***p < 0.001.

In contrast, FTT-10, FPE, and TMT-A did not contribute uniquely to HST once other predictors were controlled (all *p* ≥ 0.08). These results indicate that beyond shared variance, handwriting speed in this age group depends on efficient manual dexterity, sustained motor tempo, flexible executive control, and visuospatial working memory capacity, rather than on short-burst tapping speed or basic visual scanning alone (Table 3, Panel A).

#### 3.3.2 Model 2: predictors of handwriting legibility (HLS)

A second regression model predicting HLS handwriting legibility (higher scores = poorer legibility) from the same set of predictors explained 46.8% of the variance (*R*^2^ = 0.468, adjusted *R*^2^ = 0.437, *F* (7, 121) = 15.21, *p* < 0.001) (Table 3, Panel B). Three predictors were independently associated with legibility. Poorer manual dexterity (longer Grooved Pegboard times) was associated with poorer legibility [*B* = 0.059, 95% CI (0.029, 0.088), *t* = 3.89, *p* < 0.001]. Greater kinesthetic errors (higher FPE) predicted higher HLS scores [*B* = 0.105, 95% CI (0.025, 0.185), *t* = 2.61, *p* = 0.010], indicating less precise handwriting. Poor executive flexibility (longer TMT-B completion times) was also linked to poor legibility [*B* = 0.064, 95% CI (0.038, 0.091), *t* = 4.80, *p* < 0.001].

In this model, motor speed (FTT-10, FTT-30), TMT-A, and Corsi span were not significant independent predictors of HLS (all *p* ≥ 0.118), despite some bivariate associations. Taken together, these findings suggest that legibility is primarily constrained by fine-motor coordination, proprioceptive precision, and higher-order executive control rather than by raw tapping speed or visuospatial working memory, once other factors are considered (Table 3, Panel B).

#### 3.3.3 Summary across models

Across both models, executive flexibility (TMT-B) emerged as a common predictor of handwriting fluency and legibility, whereas sustained motor speed and visuospatial working memory were uniquely related to fluency, and kinesthetic differentiation was uniquely related to legibility. The modest negative correlation between HST and HLS (*r* = −0.27) is consistent with a partial speed–legibility trade-off, but the distinct predictor profiles indicate that speed and legibility are not simply opposite ends of a single continuum and instead reflect overlapping yet dissociable constraints.

We note that the comparatively high explained variance is likely supported by (i) the focused age range and (ii) the conceptual overlap among motor and executive predictors. Although some predictors (e.g., motor speed in the legibility model) showed bivariate associations, their unique effects diminished when shared variance was accounted for in simultaneous regression. Thus, non-significance in the multivariate models should not be interpreted as irrelevance, but rather as an indication that the predictor does not explain additional variance beyond correlated constructs.

## 4 Discussion

This cross-sectional study investigated the distinct and shared motor–cognitive determinants of handwriting proficiency in girls aged 10–12 years, with a focus on both fluency and legibility. Three primary observations were made in this study. First, a modest yet significant speed–legibility trade-off was noted (HST–HLS *r* = −0.27), aligning with models that propose partially separable mechanisms for fluency versus legibility (Frikha et al., 2025; Sumner et al., 2014; Gosse et al., 2021). Second, handwriting fluency (HST) was independently predicted by manual dexterity (Grooved Pegboard), sustained motor speed (FTT-30), executive flexibility (TMT-B), and visuospatial working memory (Corsi span), collectively accounting for approximately half of the observed variance (*R*^2^ = 0.520). Third, legibility (HLS; higher scores indicate poorer legibility) was uniquely predicted by manual dexterity, kinesthetic differentiation (force production error), and executive flexibility, explaining nearly half of the variance (*R*^2^ = 0.468). Collectively, these findings suggest that fluency depends on efficient and sustained motor output supported by executive control and visuospatial working memory, whereas legibility is more strongly influenced by fine motor precision and proprioceptive fidelity, with executive flexibility serving as a shared correlate, consistent with its unique contribution in both regression models.

An additional interpretative possibility is that part of the variance observed in English handwriting reflects transfer from Arabic L1 handwriting experience. Cross-script studies suggest that some graphomotor skills can transfer across alphabets, whereas biscriptual evidence also indicates script-specific motor reorganisation linked to the formal properties of the practiced scripts. Consequently, the present findings are likely to reflect both shared graphomotor resources and script-specific constraints rather than purely English-specific mechanisms (Asselborn et al., 2021; Alhaddad et al., 2023; Alhaddad et al., 2024).

### 4.1 Speed-legibility dynamics

The observed negative correlation between speed and legibility corroborates previous research indicating that the rate of production and graphic quality do not progress in tandem during late childhood (Sumner et al., 2014; Gosse et al., 2021). The correlation magnitude in the current study (*r* = −0.27) aligns with a small-to-moderate effect size, suggesting a partial rather than an absolute trade-off. Theories of motor control in handwriting (van Galen, 1991) and neurocognitive models (Planton et al., 2013) converge on the notion that stroke timing and shape are partially dissociable processes that are coordinated by executive systems under time constraints. From an applied perspective, this indicates that increasing writing speed without sufficient automatisation may strain monitoring resources and hinder the precise adjustments necessary for consistent letter formation, thereby reducing legibility (Feder and Majnemer, 2007; Santangelo and Graham, 2016). Concurrently, the modest correlation suggests that both speed and legibility can be enhanced simultaneously when the underlying motor and cognitive limitations are addressed rather than being inherently opposed. In this discussion, Gosse et al. (2021) is interpreted as evidence that speed and quality do not develop in perfect synchrony, whereas Planton et al. (2013) is used more cautiously to support partially dissociable neural contributions to handwriting rather than a directly observed behavioral trade-off.

### 4.2 Determinants of fluency: dexterity, sustained motor speed, executive function, and working memory

The finding that Grooved Pegboard performance and sustained tapping speed (FTT-30) emerged as independent predictors of HST aligns with the notion that both spatial coordination and temporal stability are fundamental components of proficient writing (Prunty et al., 2013; Tseng and Chow, 2000). The lack of a unique contribution from the short-burst tapping measure (FTT-10) once FTT-30 was considered suggests that continuous motor tempo, rather than merely ballistic tapping capacity, is more pertinent to classroom L2 handwriting, which necessitates sustained output over extended periods rather than isolated, explosive actions.

In terms of cognitive processes, both the TMT-B and Corsi span independently account for variance in fluency. Executive flexibility likely facilitates rapid alternation among letters and letter groups, minimises errors, and enables efficient adaptation to local demands, such as spacing and alignment. Concurrently, visuospatial working memory may support spatial layout demands (e.g., spacing and alignment) and temporary sequencing during transcription, which can facilitate fluent output under time constraints (Diamond, 2013; Salas and Silvente, 2020). McCutchen (1996) is relevant here at the broader level of resource allocation in writing, insofar as limited working-memory capacity can constrain transcription when lower-level processes are not sufficiently automatised. These findings are consistent with dual-constraint theories, which propose that automatised motor programs liberate executive and working memory resources for higher-level planning and real-time monitoring (Medwell and Wray, 2014; Downing and Caravolas, 2023). Furthermore, they support the perspective that handwriting speed is not solely a motor phenomenon, but rather an emergent property resulting from the interaction between motor execution and cognitive control systems.

### 4.3 Determinants of legibility: dexterity, kinesthetic differentiation, and executive flexibility

In contrast, legibility is predominantly associated with manual dexterity, kinesthetic differentiation, and executive flexibility. The correlation between suboptimal performance on the Grooved Pegboard test and elevated HLS scores aligns with existing evidence that fine motor coordination is fundamental to stroke geometry, spacing, and baseline consistency, all of which contribute to discernible readability (Prunty et al., 2013). The distinct contribution of force production error (FPE) indicates that proprioceptive precision, defined as the ability to accurately reproduce target forces, may aid in stabilising pen pressure and pen–paper contact, thereby facilitating more consistent letter formation. Previous research has highlighted the significance of kinesthetic differentiation ability in movement quality and postural control (Raczek et al., 2003; Prunty et al., 2020), and the current findings extend this understanding to handwriting legibility in typically developing children.

Executive flexibility, as measured by the TMT-B, has been identified as a significant predictor of both fluency and legibility, highlighting the critical role of top-down regulation. Children who completed the TMT-B more rapidly, indicative of superior set shifting and cognitive control—tended to write not only with greater speed but also with enhanced legibility. This finding aligns with evidence that executive functions support goal maintenance and adaptive adjustments during complex transcription tasks under time constraints (Diamond, 2013; Sumner et al., 2014). Within developmental samples, executive functions have been shown to predict transcription skills and written expressions, even when motor skills are considered. The present data corroborate this literature by emphasising TMT-B as a key, shared correlate across handwriting outcomes.

### 4.4 Educational and clinical implications

The distinct predictor profiles for fluency and legibility suggest that a uniform approach to handwriting remediation may not be effective. For late primary school girls whose primary challenge is fluency, interventions should focus on the automatisation of fundamental motor patterns, such as graded fine-motor drills, the training of sustained tempo through paced copying tasks with progressively increasing duration, and exercises that support executive sequencing and visuospatial memory, such as copying short-letter strings or words from memory. In contrast, for late primary school girls who primarily experience legibility issues, the emphasis should be on precision-oriented tasks, such as graded Grooved Pegboard-like activities, pressure calibration, and proprioceptive training, including tracing with varied force and feedback, and executive function-oriented strategies to maintain consistent quality under time constraints (Feder and Majnemer, 2007; Santangelo and Graham, 2016). In conventional classroom settings, these principles may be implemented through concise, focused “transcription warm-ups” integrated at the commencement of writing lessons (Frikha et al., 2025). These warm-ups would combine brief motor exercises with explicit self-monitoring prompts, such as assessments of speed and neatness. Educators might also consider adjusting expectations regarding speed versus legibility based on the specific demands of the task, such as rapid note-taking compared to final written products, acknowledging that the optimal balance may vary across different instructional activities.

### 4.5 Strengths and limitations

The strengths of this study include the relatively large and age-homogeneous sample, the employment of validated handwriting measures (HST, HLS), and a comprehensive multidomain predictor battery that includes fine-motor execution, proprioception, executive flexibility, and visuospatial working memory. The application of multivariate regression models enabled us to distinguish unique contributions from shared ones, thereby advancing beyond simple bivariate correlations. Furthermore, the focus on a narrow age range facilitates a clearer interpretation of the handwriting demands of late primary school students.

This study had several limitations. First, the cross-sectional design limits the ability to draw causal inferences; it remains unclear whether enhancing dexterity or executive flexibility would directly improve L2 handwriting, as only associations are observed. Second, the sample was confined to girls, which enhances internal homogeneity but restricts generalisability to boys and mixed-gender classrooms (Feder and Majnemer, 2007). The debate on sex differences in motor and literacy development persists, and future research should explicitly compare patterns across sexes. Accordingly, throughout the manuscript we refer to “girls” rather than “children” when drawing inferences from our data. Additionally, English (L2) proficiency was not directly assessed; therefore, the present findings should be interpreted as predictors of handwriting performance in an English-medium schooling context rather than as evidence of L2-specific mechanisms. Because age was not statistically modelled as a covariate within the restricted 10–12-year band, residual developmental variability may still have contributed to the observed associations. The present Arabic-English schooling context was also English-dominant rather than exposure-balanced, which limits generalisability to more symmetrical forms of bilingual/biscriptual experience. Third, global outcome measures (HST and HLS) were employed instead of high-resolution digital kinematic measures; tablet-based assessments could yield more detailed insights into stroke dynamics, pen pressure variability, and online correction processes (Planton et al., 2013). Likewise, the finger-tapping task did not provide a spatial accuracy metric, so our motor-speed indicator captured tempo but not spatial precision. Although predictor tasks were counterbalanced, they were not fully randomized, which may have reduced ecological variability in task sequencing. Fourth, although the predictor set encompassed key motor and executive constructs, broader language or literacy measures (e.g., reading fluency, spelling, orthographic coding) were not included, which may mediate or moderate the relationships between motor and executive skills and writing performance in ecologically valid composition tasks (McCutchen, 1996; Salas and Silvente, 2020). Phonological working memory should also be incorporated in future studies, especially because L2 transcription may draw on both visuospatial and phonological buffering. Finally, while the sample size was adequate for the number of predictors, it still constrains more complex modelling (e.g., structural equation modelling or multi-group analyses).

### 4.6 Future directions

Future research should extend beyond cross-sectional associations to empirically test causal hypotheses using longitudinal and intervention designs. Randomized controlled trials could assess whether targeted training in fine-motor dexterity, kinesthetic differentiation, or executive flexibility results in measurable improvements in fluency and legibility and whether these improvements extend to broader academic outcomes (e.g., essay quality and written exams). The integration of kinematic tablet measures with cognitive tasks may facilitate the identification of specific bottlenecks (e.g., temporal irregularity and excessive pressure variability) and enhance individualised intervention plans. Comparative studies of paper and digital handwriting environments could further elucidate how different sensory feedback channels (haptic vs. visual) influence the balance between speed and legibility (Planton et al., 2013; Marano et al., 2025). Finally, incorporating motor, executive, and language variables into multilevel or structural models could provide a more comprehensive framework for understanding the development of L2 transcription skills and their constraints on writing compositions. In addition, multivariate/structural approaches could formally model the correlation between fluency and legibility and test whether predictors exert differential effects across these outcomes. Future research should also model age and/or school grade explicitly, include phonological alongside visuospatial working-memory measures, derive motor tasks that capture both tempo and spatial accuracy, compare counterbalanced versus fully randomized task orders, and examine more balanced versus English-dominant Arabic-English biscriptual settings.

## 5 Conclusion

In conclusion, L2 handwriting proficiency in late primary school girls in an English-medium schooling context is indicative of the interaction between various motor and cognitive systems, rather than a singular underlying trait. Fluency is contingent upon manual dexterity, sustained motor tempo, and support of executive and visuospatial working memory, whereas legibility relies more heavily on fine-motor precision, proprioceptive accuracy, and executive flexibility. The presence of only a modest speed-legibility trade-off suggests that both dimensions can be enhanced simultaneously if their underlying constraints are addressed. Customising assessment and instruction for these facet-specific profiles while conserving cognitive resources for higher-level writing processes may provide a principled approach to improving both the speed and quality of L2 handwritten work in actual classroom settings.

## Data Availability

The raw data supporting the conclusions of this article will be made available by the authors, without undue reservation.

## Footnotes

1
https://apps.apple.com/us/app/speedy-tappers/id1271574174

2
https://www.memorylosstest.com/corsi-block-tapping-test/

## References

[ref1] Afonso O. Suárez-Coalla P. González-Martín N. Cuetos F. (2018). The impact of word frequency on peripheral processes during handwriting: a matter of age. Q. J. Exp. Psychol. 71, 695–703. doi: 10.1080/17470218.2016.1275713, 28054498

[ref2] Alhaddad G. Danna J. Younes-Harb C. Velay J.-L. Longcamp M. (2023). Effects of biscriptuality on graphomotor coordination dynamics. J. Exp. Psychol. Hum. Percept. Perform. 49, 177–187. doi: 10.1037/xhp0001075, 36455050

[ref3] Alhaddad G. Dione M. Danna J. Alario F.-X. Honnorat A. Velay J.-L. . (2024). Writing in two different scripts promotes fine motor control. Cortex 179, 247–260. doi: 10.1016/j.cortex.2024.07.016, 39213777

[ref4] Altheneyan A. Boayrid N. F. (2019). Writing errors among Arab EFL learners: a review of literature. Int. J. Linguist. 11, 319–329. doi: 10.5296/ijl.v11i5.15294

[ref5] Araújo S. Domingues M. Fernandes T. (2022). From hand to eye: a meta-analysis of the benefit from handwriting training in visual graph recognition. Educ. Psychol. Rev. 34, 1577–1612. doi: 10.1007/s10648-021-09651-4

[ref6] Asselborn T. Johal W. Tleubayev B. Zhexenova Z. Dillenbourg P. McBride C. . (2021). The transferability of handwriting skills: from the Cyrillic to the Latin alphabet. NPJ Sci. Learn. 6:6. doi: 10.1038/s41539-021-00084-w, 33623040 PMC7902616

[ref7] Barnett A. L. Prunty M. Rosenblum S. (2018). Development of the handwriting legibility scale (HLS): a preliminary examination of reliability and validity. Res. Dev. Disabil. 72, 240–247. doi: 10.1016/j.ridd.2017.11.013, 29223112

[ref300] Bonneton-Botté N. Miramand L. Bailly R. Pons C. (2023). Teaching and rehabilitation of handwriting for children in the digital age: issues and challenges. Children (Basel, Switzerland), 10, 1096. doi: 10.3390/children10071096, 37508593 PMC10378357

[ref8] Bonafede C. van der Merwe E. (2023). Kinesthetic coordination abilities in 6-year-old children: school quintile, gender, and hand dominance differences. Int. J. Early Child. 56, 277–295. doi: 10.1007/s13158-023-00350-5, 36844145 PMC9937861

[ref10] Connelly V. Dockrell J. E. Barnett J. (2005). The slow handwriting of undergraduate students constrains overall performance in exam essays. Educ. Psychol. 25, 99–107. doi: 10.1080/0144341042000294912

[ref11] Cook V. J. Bassetti B. (2005). Second Language Writing Systems. Clevedon: Multilingual Matters.

[ref12] Corsi P. M. (1973) *Human memory and the medial temporal region of the brain*. (doctoral thesis). ProQuest Information & Learning, Ann Arbor, MI, United States

[ref13] Diamond A. (2013). Executive functions. Annu. Rev. Psychol. 64, 135–168. doi: 10.1146/annurev-psych-113011-143750, 23020641 PMC4084861

[ref14] Downing C. Caravolas M. (2023). Handwriting legibility and fluency and their patterns of concurrent relations with spelling, graphomotor, and selective attention skills. J. Exp. Child Psychol. 236:105756. doi: 10.1016/j.jecp.2023.105756, 37544070

[ref15] Feder K. P. Majnemer A. (2007). Handwriting development, competency, and intervention. Dev. Med. Child Neurol. 49, 312–317. doi: 10.1111/j.1469-8749.2007.00312.x, 17376144

[ref16] Frikha M. Alsalman Z. A. Lenoir M. (2025). Differential impact of stretching modalities on handwriting proficiency, executive function, and working memory in school-aged girls. Read. Writ. doi: 10.1007/s11145-025-10712-1

[ref18] Giustino V. Patti A. Petrigna L. Figlioli F. Thomas E. Costa V. . (2023). Manual dexterity in school-age children measured by the grooved pegboard test: evaluation of training effect and performance in dual-task. Heliyon 9:e18327. doi: 10.1016/j.heliyon.2023.e18327, 37539174 PMC10395525

[ref19] Gosse C. Parmentier M. Van Reybroeck M. (2021). How do spelling, handwriting speed, and handwriting quality develop during primary school? Cross-classified growth curve analysis of children’s writing development. Front. Psychol. 12:685681. doi: 10.3389/fpsyg.2021.685681, 34367011 PMC8343101

[ref20] Graham S. Weintraub N. (1996). A review of handwriting research: progress and prospects from 1980 to 1994. Educ. Psychol. Rev. 8, 7–87. doi: 10.1007/bf01761831

[ref21] Haake L. (2025). Does the relationship between executive functions and L2 writing depend on language proficiency? J. Writ. Res. 17, 115–153. doi: 10.17239/jowr-2025.17.01.05

[ref22] Haleem A. Javaid M. Qadri M. A. Rajiv Suman R. (2022). Understanding the role of digital technologies in education: a review. Sustain. Operat. Comp. 3, 275–285. doi: 10.1016/j.susoc.2022.05.004

[ref24] Heimhofer C. Neumann A. Odermatt I. Bachinger M. Wenderoth N. (2024). Finger-specific effects of age on tapping speed and motor fatigability. Front. Hum. Neurosci. 18:1427336. doi: 10.3389/fnhum.2024.1427336, 39386279 PMC11461208

[ref9001] Kent S. Wanzek J. Petscher Y. Al Otaiba S. Kim Y. S. (2014). Writing fluency and quality in kindergarten and first grade: The role of attention, reading, transcription, and oral language. Read. Writ, 27, 1163–1188. doi: 10.1007/s11145-013-9480-1 25132722 PMC4133358

[ref27] Kormos J. (2023). The role of cognitive factors in second language writing and writing to learn a second language. Stud. Second. Lang. Acquis. 45, 622–646. doi: 10.1017/S0272263122000481

[ref28] Lopez C. Vaivre-Douret L. (2021). Influence of visual control on the quality of graphic gesture in children with handwriting disorders. Sci. Rep. 11:23537. doi: 10.1038/s41598-021-02969-7, 34876643 PMC8651655

[ref29] Marano G. Kotzalidis G. D. Lisci F. M. Anesini M. B. Rossi S. Barbonetti S. . (2025). The neuroscience behind writing: handwriting vs. typing—who wins the battle? Life 15:345. doi: 10.3390/life15030345, 40141690 PMC11943480

[ref30] Mathiowetz V. Wiemer D. M. Federman S. M. (1986). Grip and pinch strength: norms for 6- to 19-year-olds. Am. J. Occup. Ther. 40, 705–711. doi: 10.5014/ajot.40.10.705, 3777107

[ref31] Maurer M. N. (2024). Correlates of early handwriting: differential patterns for girls and boys. Early Educ. Dev. 35, 843–858. doi: 10.1080/10409289.2023.2244349

[ref32] Maurer M. N. Truxius L. Sägesser Wyss J. Eckhart M. (2023). From scribbles to script: Graphomotor skills' impact on spelling in early primary school. Children 10:1886. doi: 10.3390/children10121886, 38136088 PMC10741596

[ref33] McCutchen D. (1996). A capacity theory of writing: working memory in composition. Educ. Psychol. Rev. 8, 299–325. doi: 10.1007/bf01464076

[ref34] Medwell J. Wray D. (2013). Handwriting automaticity: the search for performance thresholds. Lang. Educ. 28, 34–51. doi: 10.1080/09500782.2013.763819

[ref35] Medwell J. Wray D. (2014). “Handwriting and writing,” in The Routledge International Handbook of English, Language and Literacy Teaching, eds. Wyse D. Andrews R. Hoffman J. (London: Routledge).

[ref36] Merker B. Podell K. (2011). “Grooved pegboard test,” in Encyclopedia of Clinical Neuropsychology, eds. Kreutzer J. S. DeLuca J. Caplan B. (New York: Springer).

[ref37] Odlin T. (2003). “Cross-linguistic influence,” in The Handbook of Second Language Acquisition, eds. Doughty C. J. Long M. H. (Hoboken: Wiley), 436–486.

[ref38] Ose Askvik E. Weel F. R. van der Meer A. L. H. (2020) The importance of cursive handwriting over typewriting for learning in the classroom: a high-density EEG study of 12-year-old children and young adults Front. Psychol. 11:1810 doi: 10.3389/fpsyg.2020.01810 32849069 PMC7399101

[ref39] Planton S. Jucla M. Roux F. E. Demonet J. F. (2013). The “handwriting brain”: a meta-analysis of neuroimaging studies of motor versus orthographic processes. Cortex 49, 2772–2787. doi: 10.1016/j.cortex.2013.05.011, 23831432

[ref40] Potgieser A. R. van der Hoorn A. de Jong B. M. (2015). Cerebral activations related to writing and drawing with each hand. PLoS One 10:e0126723. doi: 10.1371/journal.pone.0126723, 25955655 PMC4425548

[ref41] Prunty M. M. Barnett A. L. Wilmut K. Plumb M. S. (2013). Handwriting speed in children with developmental coordination disorder: are they really slower? Res. Dev. Disabil. 34, 2927–2936. doi: 10.1016/j.ridd.2013.06.005, 23816628

[ref42] Prunty M. M. Pratt A. Raman E. Simmons L. Steele-Bobat F. (2020). Grip strength and pen pressure are not key contributors to handwriting difficulties in children with developmental coordination disorder. Br. J. Occup. Ther. 83, 387–396. doi: 10.1177/0308022619885046

[ref43] Raczek J. Mynarski W. Ljach W. (2003). Kształtowanie i Diagnozowanie Koordynacyjnych Zdolności Motorycznych: Podręcznik dla Nauczycieli, Trenerów i Studentów. Katowice: The Academy of Physical Education in Katowice.

[ref44] Reitan R. M. Wolfson D. (2001). “The Halstead–Reitan neuropsychological test battery: research findings and clinical application,” in Specific Learning Disabilities and Difficulties in children and Adolescents: Psychological Assessment and Evaluation, eds. Kaufman A. S. Kaufman N. L. (Cambridge: Cambridge University Press), 309–346.

[ref45] Richardson J. T. E. (2007). Measures of short-term memory: a historical review. Cortex 43, 635–650. doi: 10.1016/S0010-9452(08)70493-3, 17715798

[ref9002] Rosenblum S. Amit Ben-Simhon H. Meyer S. Gal E. (2019). Predictors of handwriting performance among children with autism spectrum disorder. Res Autism Spectr Disord. 60, 16–24. doi: 10.1016/j.rasd.2019.01.002

[ref47] Rosenblum S. Parush S. Weiss P. L. (2003). Computerized temporal handwriting characteristics of proficient and non-proficient handwriters. Am. J. Occup. Ther 57, 129–138. doi: 10.5014/ajot.57.2.129, 12674304

[ref48] Salas N. Silvente S. (2020). The role of executive functions and transcription skills in writing: a cross-sectional study across 7 years of schooling. Read. Writ. 33, 877–905. doi: 10.1007/s11145-019-09979-y

[ref49] Santangelo T. Graham S. (2016). A comprehensive meta-analysis of handwriting instruction. Educ. Psychol. Rev. 28, 225–265. doi: 10.1007/s10648-015-9335-1

[ref50] Schatz P. (2011). “Finger tapping test,” in Encyclopedia of Clinical Neuropsychology, eds. Caplan B. Kreutzer J. S. DeLuca J. (New York: Springer), 1050–1051.

[ref51] Semeraro C. Coppola G. Cassibba R. Lucangeli D. (2019). Teaching of cursive writing in the first year of primary school: effect on reading and writing skills. PLoS One 14:e0209978. doi: 10.1371/journal.pone.0209978, 30730894 PMC6366728

[ref52] Simfukwe C. Baek M. J. Kim S. An S. S. (2020). Digital trail making test-black and white: for android and iOS. Alzheimers Dement. 16:e037886. doi: 10.1002/alz.037886

[ref53] Skar G. B. Graham S. Huebner A. R. (2023). Efficacy for writing self-regulation, attitude toward writing, and quality of second grade students’ writing. Front. Psychol. 14:1265785. doi: 10.3389/fpsyg.2023.1265785, 37915520 PMC10616524

[ref55] Stuart N. Zoia S. Biancotto M. Barnett A. L. (2024). The handwriting legibility scale: a language and age extension for students with and without specific learning difficulties. J. Mot. Learn. Dev. 12, 610–634. doi: 10.1123/jmld.2023-0029

[ref56] Sumner E. Connelly V. Barnett A. L. (2012). Children with dyslexia are slow writers because they pause more often and not because they are slow at handwriting execution. Read. Writ. 26, 991–1008. doi: 10.1007/s11145-012-9403-6

[ref57] Sumner E. Connelly V. Barnett A. L. (2014). The influence of spelling ability on handwriting production: children with and without dyslexia. J. Exp. Psychol. Learn. Mem. Cogn. 40, 1441–1447. doi: 10.1037/a0035785, 24548322

[ref58] Tolle K. A. Rahman-Filipiak A. M. Hale A. C. Kitchen Andren K. A. Spencer R. J. (2020). Grooved pegboard test as a measure of executive functioning. Appl. Neuropsychol. Adult 27, 414–420. doi: 10.1080/23279095.2018.1559165, 30734576

[ref59] Tseng M. H. Chow S. M. (2000). Perceptual-motor function of school-age children with slow handwriting speed. Am. J. Occup. Ther. 54, 83–88. doi: 10.5014/ajot.54.1.83, 10686631

[ref9003] Vaivre-Douret L. Lopez C. Dutruel A. Vaivre S. (2021). Phenotyping features in the genesis of pre-scriptural gestures in children to assess handwriting developmental levels. Sci. rep. 11:731. doi: 10.1038/s41598-020-79315-w, 33436668 PMC7804314

[ref60] van Galen G. P. (1991). Handwriting: issues for a psychomotor theory. Hum. Mov. Sci. 10, 165–191. doi: 10.1016/0167-9457(91)90003-g

[ref61] Vasylets O. Marín J. (2021). The effects of working memory and L2 proficiency on L2 writing. J. Second. Lang. Writ. 52:100786. doi: 10.1016/j.jslw.2020.100786

[ref62] Vico R. Martin J. Gonzalez M. (2023). Functional assessment of handwriting among children: a systematic review of the psychometric properties. Am. J. Occup. Ther. 77. doi: 10.5014/ajot.2023.050174, 37877571

[ref63] Wallen M. Bonney M. A. Lennox L. (2006). The handwriting speed test. Aust. Occup. Ther. J. 53, 141–141. doi: 10.1111/j.1440-1630.2006.00571.x

[ref64] Wallen M. Mackay S. (1999). Test-retest, interrater, and intrarater reliability, and construct validity of the handwriting speed test in year 3 and year 6 students. Phys. Occup. Ther. Pediatr. 19, 29–42. doi: 10.1300/J006v19n01_03

[ref65] Wilcox G. Nordstokke D. (2022). Pediatric co-norms for finger tapping, grip strength, and grooved pegboard in a community sample. J. Int. Neuropsychol. Soc. 28, 85–93. doi: 10.1017/S1355617721000175, 33749572

[ref66] Yang Y. Tam F. Graham S. J. Sun G. Li J. Gu C. . (2020). Men and women differ in the neural basis of handwriting. Hum. Brain Mapp. 41, 2642–2655. doi: 10.1002/hbm.24968, 32090433 PMC7294055

